# Gut microbe-derived metabolite indole-3-carboxaldehyde alleviates atherosclerosis

**DOI:** 10.1038/s41392-023-01613-2

**Published:** 2023-10-04

**Authors:** Yijing Lu, Wenlong Yang, Zhiyong Qi, Rifeng Gao, Jing Tong, Tingwen Gao, Yin Zhang, Aijun Sun, Shuning Zhang, Junbo Ge

**Affiliations:** 1 Institutes of Biomedical Sciences, Fudan University, Shanghai, China; 2 Department of Cardiology, Zhongshan Hospital, Fudan University, Shanghai, China; 3 Department of Cardiology, Tongji Hospital, School of Medicine, Tongji University, Shanghai, China

**Keywords:** Cardiology, Predictive markers

**Dear Editor**,

Indole-3-carboxaldehyde (ICA) is a gut microbe-derived tryptophan metabolite. Here, we explored the role of ICA in atherosclerosis.

Plasma tryptophan metabolites were measured in healthy individuals and patients with atherosclerosis (Supplementary Table S1). The latter group showed disturbed tryptophan metabolism, while no difference was observed in tryptophan levels between the two groups (Fig. 1a). ICA concentration is lower in the patients group (Fig. 1b), consistent with previous clinical studies.^1^ The concentration of other metabolites of kynurenine or serotonin pathway showed no variations between the control and the patients (Supplementary Fig. 1a).

**Fig. 1:**
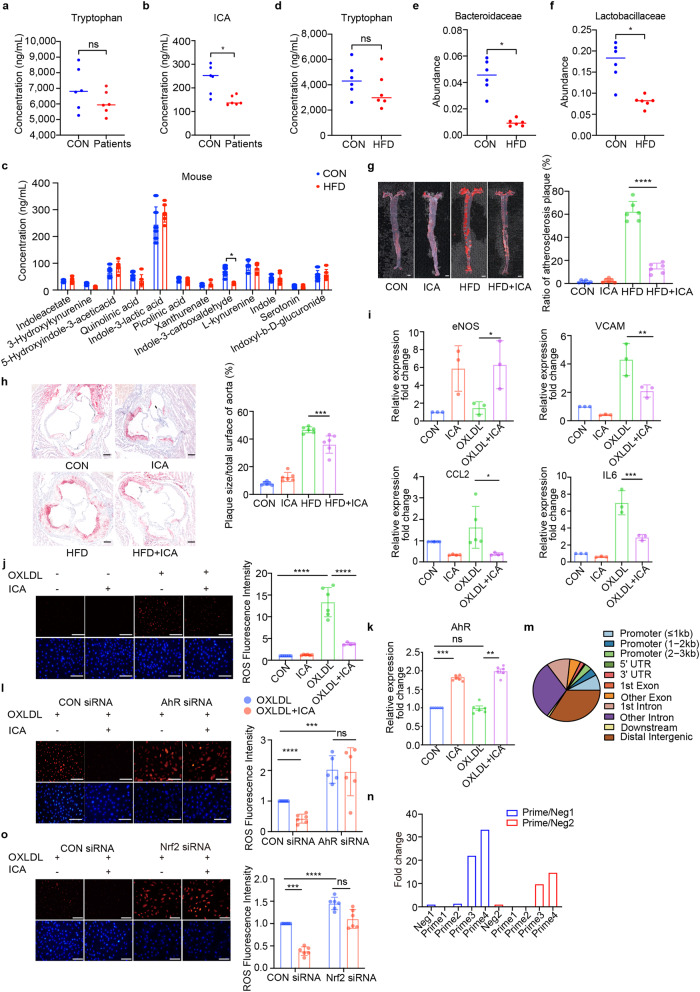
Gut microbe-derived metabolite indole-3-carboxaldehyde alleviates atherosclerosis. **a** Tryptophan concentration in patients with atherosclerosis and healthy controls. **b** ICA concentrations in patients with atherosclerosis and healthy controls. **c** The concentration of plasma tryptophan metabolites in the high-fat diet and control groups (*n* = 6). **d** Plasma tryptophan levels in the Western diet and control groups. **e** *Bacteroidaceae* levels in HFD-fed and control groups. **f** *Lactobacillaceae* levels in HFD-fed and control groups. **g** Representative images of Oil Red O staining of the atherosclerotic plaque area in the whole aorta (*n* = 6, scale bar: 1 mm). Quantitative analysis of the atherosclerotic plaque area in the whole aorta. Data are presented as mean ± SD and were analyzed using one-way ANOVA and Tukey’s multiple comparison test. **h** Representative images of Oil Red O staining of the aortic sinus lesion area (*n* = 10, scale bar: 100 μm). Data are presented as means ± SD. Data were analyzed using an unpaired two-tailed Student *t*-test. **i** Real-time polymerase chain reaction analysis of the endothelial function associated genes eNOS, VCAM, CCL2, and IL6 in endothelial cells treated with ox-LDL (100 mg/mL) and ICA (100 nmol) (*n* = 4–6 per group). **j** DHE and DAPI staining of HUVEC treated with ox-LDL or ICA, respectively (*n* = 6, scale bar: 100 μm). Quantification of DHE fluorescence intensity. Data are presented as mean ± SEM and statistical significance was determined by one-way ANOVA with Tukey’s multiple comparisons test. **k** Expression of AhR in HUVEC treated with ICA (*n* = 4 per group). **l** DHE and DAPI staining of HUVEC transfected with control siRNA or AhR siRNA treated with ox-LDL and ICA, respectively (*n* = 6, scale bar: 100 μm). Quantification of the DHE fluorescence intensity. Data are presented as mean ± SEM, and *p*-values were determined by two-way ANOVA with Tukey’s multiple comparison test. **m** Sector graphs of different functional element regions in the genome integrated with AhR. AhR binds to the promoter region of Nrf2, **n** as supported by the results of chip-qPCR. Data are presented as mean ± SEM, and statistical significance was determined using an unpaired two-tailed Student *t*-test. **o** DHE and DAPI staining of HUVEC cells transfected with control siRNA or Nrf2 siRNA treated with ox-LDL and ICA, respectively (*n* = 6, scale bar: 100 μm). Quantification of the DHE fluorescence intensity. Data are presented as mean ± SEM, and *p*-values were determined by two-way ANOVA and Tukey’s multiple comparison test. (*p < 0.05, **p ≤ 0.01, ***p ≤ 0.001, ****p ≤ 0.0001)

Our rodent model depicted a pattern in consistency with our human data, where the high-fat diet (HFD) group exhibited a lower level of ICA, though there were no differences in tryptophan concentration (Fig. 1c, d). ICA administration raised ICA plasma level (Supplementary Fig. 1b). Since the tryptophan metabolism partially depends on the activity of gut microbes,^2^ we employed metagenomics to study the gut microbes. The abundance of *Bacteroidaceae* and *Lactobacillaceae* in the HFD group was lower than that in the control group (Fig. 1e, f). These bacteria have been proven to produce tryptophanase metabolizing tryptophan into ICA. The lack of these gut microbes prevented the metabolism of tryptophan to ICA.

We then considered how ICA would affect atherosclerosis. Oil Red O staining revealed that plaques in the HFD + ICA group were dramatically smaller in the whole aorta en face (HFD vs. HFD + ICA: *p* < 0.0001, Fig. 1g). The total plaque area decreased by 40%. The plaque area at the aortic roots in the HFD + ICA group was 20% smaller than that in the HFD group. (*p* = 0.0005, Fig. 1h). Taken together, ICA minimized the plaque area and delayed atherosclerosis progression.

Previous studies have proved ICA’s role in enhancing the epithelial barrier and anti-inflammatory activity in the intestinal tract.^3^ Here we instead focus on the role of ICA on endothelial cells. We found that ICA did not affect the RNA levels of intercellular cell adhesion molecule (ICAM) and endothelin (ET-1) but did increase endothelial nitric oxide synthase (eNOS) transcription levels (supplementary Fig. 2a, b and Fig. 1i). Furthermore, ICA reduced gene expression of vascular cell adhesion molecule (VCAM), C–C motif chemokine ligand 2 (CCL2), and interleukin- 6 (IL6) (Fig. 1i). Besides, ICA reduced the total reactive oxygen species (ROS) level, induced by ox-LDL, by 70% (*p* < 0.0001, Fig. 1j). Furthermore, through HE staining on plaques, we observed that ICA prevented the plaques development with less necrotic cores and decreased plaque thickness. With the supplement of ICA, less inflammatory cells were found in the plaques (Supplementary Fig. 2c).

Molecules containing aromatic hydrocarbons are potential ligands for AhR. ICA increased AhR gene expression (Fig. 1k) and downstream genes of AhR (Supplementary Fig. 3a–c), demonstrating AhR activation by ICA. We also detected increased AhR expression in HFD + ICA group (Supplementary Fig. 3d). Furthermore, after AhR knockdown (Supplementary Fig. 3e, f), gene expression of VCAM CCL2 and IL6 increased, whereas eNOS transcription levels reduced (Supplementary Fig. 3g, h). No difference was observed in ICAM and ET-1 levels (Supplementary Fig. 3i). Moreover, ICA no longer controlled ROS levels (Fig. 1l).

To further study how ICA reduces ROS through AhR, we first screened the downstream genes of AhR by cleavage under targets and mentation (CUT&Tag) analysis (Fig. 1m). Results showed, compared to control, AhR mostly binds to the promoter region in ICA-treated HUVEC cells, among which NF-E2-related Factor (Nrf2) raised our attention. In our ChIP-PCR analysis (Fig. 1n), as confirmed by a luciferase reporter assay (supplementary Fig. 4a, b), ICA treatment increased the transcription and expression of Nrf2 and heme oxygenase (HO-1), a downstream gene of Nrf2 (Supplementary Fig. 4c–f). When AhR was knocked down, Nrf2 and HO-1 gene expression and protein expression levels decreased (Supplementary Fig. 4g–i).

To explore whether Nrf2 participates in ICA-triggered protection, we performed Nrf2 knockdown (Supplementary Fig. 5a). Inhibition of Nrf2 reduced expression of HO-1 and AhR (Supplementary Fig. 5b, c), yet increased expression of CCL2 and IL6 (Supplementary Fig. 5d, e). ICA did not increase eNOS expression, nor did it reduce VCAM expression with Nrf2 silenced (Supplementary Fig. 5f, g). Meanwhile, the ROS level was higher than that in the control, even with ICA treatment (Fig. 1o and Supplementary Fig. 5h, i). In summary, Nrf2 was indispensable for the antioxidant activity of ICA. ICA reduced oxidative stress by promoting AhR to upregulate the Nrf2 anti-oxidation pathway.

We then knocked down HO-1 to verify the anti-oxidation role of the AhR-Nrf2 pathway (Supplementary Fig. 6a). Silence of HO-1 suppressed some actions of ICA, yet increased eNOS and ET-1 (Supplementary Fig. 6b–f), as previous study has confirmed that eNOS expression is suppressed by HO-1.^4^ No change was observed in the expression of ICAM, Nrf2 or AhR (supplementary Fig. 6g). With HO-1 silenced, the ROS level remained high in the ICA supplement group (Supplementary Fig. 6h, i). We further tested whether ICA preserved its function when ROS levels returned to normal. N-acetyl-l-cysteine (NAC) is used as an antioxidant for sequestering ROS. The NAC + ICA-treated group and the NAC group showed similar RNA expression patterns, where NAC decreased ROS levels and all inflammatory and adhesion factors decreased (supplementary Fig. 6j–l). Collectively, our results confirmed the mechanism by which ICA activates AhR binding to the Nrf2 promoter and triggers the antioxidant gene HO-1. Therefore, ICA protects HUVEC cells from inflammation and dysfunction caused by oxidative stress.

To determine the effect of ICA in vivo, we first tested the ROS level in vivo. ROS levels in the aortic root were slightly decreased in HFD + ICA group. Furthermore, ICA alleviated the oxidative stress in liver, suggesting that ICA is an effective antioxidant (Supplementary Fig. 7a). The plasma concentration of CCL2 was lower in the HFD + ICA group (Supplementary Fig. 7b). We tested the mRNA level of these genes in aortic tissues (Supplementary Fig. 7c), which showed ICA induced AhR activation in the aortic tissues and in turn increased the expression of CYP1a1. The increased expression of Nrf2 and HO-1 provided further evidence for the antioxidant effect of ICA. These results suggested that ICA regulated the AhR pathway and activated anti-oxidation and anti-inflammation in vivo.

Gut microbes are heavily involved in tryptophan digestion. When HFD gradually leads to the absence of *Bacteroidaceae* or *Lactobacillacea*, the indole pathway becomes impaired.^2^ Previous study has proved that ICA promotes the expansion of *Lactobacillus* reuteri, a group of *Lactobacillaceae*. ICA treatment probably prevents the HFD-induced change in the absence of *Bacteroidaceae* or *Lactobacillaceae*. Acting on the endothelial cells, ICA attenuates the release of pro-inflammatory cytokines and ROS levels. The effects of ICA are AhR-dependent. Activated AhR triggers the Nrf2-HO-1 pathway in endothelial cells, which reduces pro-atherosclerosis factors and ROS levels. ICA decreased ROS levels and promoted HO-1 expression in a diabetic rodent model.^5^ Our study showed that ICA decreased oxidative stress in aortic and liver tissues alike, suggesting ICA is a systematic antioxidant in vivo.

There are some limitations in the study. Clinical experiments are suggested to verify the effect of ICA. Besides, we observed a decreased expression of AhR after Nrf2 knockdown, suggesting a reciprocal regulation. Further research is needed to explore the relationship between AhR and Nrf2.

In summary, we studied the protective role of ICA in atherosclerosis. We have identified that ICA alleviates the development of atherosclerosis. We further confirmed that ICA reduces ROS levels and inflammatory factors expression in endothelial cells. This work provides another evidence that gut microbe is a potential target for atherosclerosis.

### Supplementary information

Supplementary_Materials

Gut microbe-derived metabolite indole-3-carboxaldehyde alleviates atherosclerosis

## Data Availability

The sequencing data were deposited into the Gene Expression Omnibus database under the accession number and are available at the following URL: https://www.ncbi.nlm.nih.gov/bioproject/PRJNA986702.
