# Supplementary material for: Gut microbe-derived metabolite indole-3-carboxaldehyde alleviates atherosclerosis
Source: Signal Transduct Target Ther. 2023 Oct 4;8:378. doi: 10.1038/s41392-023-01613-2 (PMC10547776; doi:10.1038/s41392-023-01613-2)
Supplement: Supplementary file 1 — Supplementary_Materials [file 41392_2023_1613_MOESM1_ESM.docx]

Supplementary Materials for

Gut microbe-derived metabolite indole-3-carboxaldehyde alleviates atherosclerosis

Yijing Lua-e†, Wenlong Yangb-e†, Zhiyong Qib-e, Rifeng Gaof, Jing Tong g, Tingwen Gaob-e, Yin Zhanga-e, Aijun Suna-e*, Shuning Zhangb-e*, Junbo Gea-e.

Correspondence to: jbge@zs-hospital.sh.cn

**This PDF file includes:**

Materials and Methods

Legends of Figures. S1 to S6

Tables S1

Materials and Methods

Human Tissue Collection

Atherosclerosis was confirmed in patients by experienced cardiothoracic surgeons and the clinical phenotype diagnosis was confirmed by standard coronary angiography at the Zhongshan Hospital Affiliated to Fudan University. The study was approved by the Ethics Committee of the Zhongshan Hospital Affiliated to Fudan University (No. 2021-656R) and performed in compliance with the Declaration of Helsinki. Blood is collected in heparin lithium-anticoagulant tubes(BD) and stored at room temperature for an hour. Centrifuge the tubes at 3000 rpm/min for15 min. Supernatant is preserved at -80℃.

Mice

All male ApoE-/- mice were obtained from Cavens at age of 8 weeks. Mice are raised in a constant temperature and humidity room with a normal circadian rhythm. All mice were divided into 6 groups. They were randomly divided into normal diets, normal diets with ICA, high-fat diets, high-fat diets with ICA (100 nmol/mL), and high-fat diets with tryptophan. High-fat diet group mice received the high-fat rodent diet with 1.25% cholesterol (D12108C). Mice in ICA, high-fat diet and ICA groups were supplied with ICA through gavage administration. All mice were raised for four months to induce atherosclerosis. Weights were measured every week. Mice were anesthetized with chloralhydrate. Blood was collected after eye removal. Plasma was obtained after 10 min 3000 rpm centrifugation at 4 °C. Fecal samples of mice were collected under aseptic conditions. The animal research protocol was approved by the Experimental Animal Ethics Committee of Fudan University. All the experimental procedures were performed according to the Guiding Principles in the Use and Care of Animals (NIH Publication No. 85–23, revised 1996).

O’oil red staining of Aortic Atherosclerotic Lesion Areas

Whole aortas were isolated and opened longitudinally for en-face staining of atherosclerotic lesions. Excess adventitial fat on the aortas was removed under a stereomicroscope and the tissue was washed in PBS. The aortas were then washed in 78% methanol for 5 s. Serial 5-mm-thick sections were obtained from the aortic root for histological analysis of atherosclerotic lesions. Staining was conducted according to the previously described protocol [12]. Plaques were measured and quantified by detecting positively stained areas (red) with ImageJ software (US National Institutes of Health, Bethesda, MD, USA).

Metagenome

The fecal gDNA (1 μg) sample was sheared into fragments. Fragmented DNA was cleaned up for end repair with freshly prepared 80% EtOH. ERP2 or ERP3 was added for repairing ends. Then 3' ends were adenylated by adding thawed ATL or ATL and incubating at 37°C and 70°C for 30 and 5 min, respectively. After adapter ligation, DNA fragments were enriched. Metagenome sequencing and analysis were conducted by OE Biotech Co., Ltd. (Shanghai, China).

Liquid Chromatography-Mass Spectrometry

The plasma (150 μL) was mixed with 2-chloro- l-phenylalanine (0.3 mg/mL) dissolved in methanol as an internal standard and the mixture was vortexed for 10 s. An ice-cold mixture of methanol and acetonitrile (2/1, v/v) was added and revortexed for 1 min. After ultrasonication at ambient temperature (25 °C to 28 °C) for 10 min, the mixture was stored at 20 °C for 30 min. The mixture was centrifuged at 13000 rmp 4°C for 15 min, the supernatant was dried in a freeze-concentration centrifugal dryer. A mixture of methanol and water (1/4, v/v) was added to the sample. Samples were centrifuged at 13000 rpm, 4 °C for 5 min after vortexing and cooling at 4°C for 2 min and filtered through 0.22 μm microfilters. The supernatants are transferred to LC vials for LC-MS analysis. QC samples were prepared by mixing aliquots of all samples to be a pooled sample.

CCL2 ELISA test

The Elisa test of CCL2 was conducted using the Mouse CCL2/MCP-1 ELISA Kit (Liankebio, EK287/2-96) according to the manufacture’s protocol.

Cell culture

HUVEC was purchased from iCell Bioscience Inc. Cells were plated as required for the specific experiments. 293T was obtained from a coworker in the lab. Concentrations of agents were 100 ng/mL for ox-LDL (Yiyuan biotechnology), and 0.1 mM for ICA (Sigma). Cells were treated with ox-LDL for 24h, and ICA was added for the second 12 h.

DHE staining for ROS detection

After 12 h of treatment, dihydroethidium (Yeason) was added to the culture medium（1:1000, v/v）for 30 min. Treated cells were then fixed in 4% formalin for 30 min. The cells were analyzed using an inverted optical microscope (ZEISS Group, Jena, Germany). The aortic tissue and liver should be immediately put in liquid nitrogen after removal, and staining should be performed in one day.

Quantitative PCR

Total mRNA was extracted using FastPure Cell/Tissue Total RNA Isolation Kit (Vazyme). cDNA was synthesized for qRT-PCR using PrimeScript™ RT Master Mix (Perfect Real Time). qRT-PCR was performed using Hieff® qPCR SYBR Green Master Mix (No Rox) (YEASEN). Primers were obtained from the Harvard primer bank and verified for specificity using primer-blast. The experiment was conducted using (Biorad CFX96). Relative expression of target genes was normalized to beta-actin and showed a fold-change in expression compared to the untreated controls following the 2−∆∆CT method.

Western blot

Cells were lysed in 1X RIPA lysis buffer with 1X protease inhibitor, 1X phosphatase inhibitor, and 1 mM PMSF added immediately before use. Protein concentrations were calculated using Bradford Protein Assay Kit. Protein was added in polyacrylamide gels according to the concentration and separated using electrophoresis and transferred to the PVDF membrane. The membrane was blocked with 5% BSA. Then the membrane was incubated with primary antibody at 4 °C overnight. The next day membranes were incubated with a secondary antibody at 4 °C for 1 h, after being washed three times with TBST. The Image j software was used to determine densitometry. Actin and the non-treated band were used to normalize target protein expression. (The primary antibodies included: AhR Antibody (CST, 83200) (1:1000); Nrf2 Antibody (Invitrogen, MA542371) (1:1000); HO-1 Antibody (CST, 26416) (1:1000)

CUT &Tag and analysis.

Make transposase following the manual of hyperactive pG-Tn5/pA-Tn5 transposase for CUT&Tag kit (Vazyme). Isolate cells and incubate with ConA Beads. Incubate with AhR antibodies (1:50) or IgG control antibody(the final concentration of antibody is 10–20 μg/mL) overnight at 4 ℃. Incubate the mixture with transposase. Add tagmentation buffer to each reaction. After 1 h, extract DNA and set up the PCR reaction. After purifying PCR product, perform the next-generation sequencing and bioinformatic analysis in Shanghai oebiotech company.

Transfections and luciferase assays

-Nrf2 promoter plasmid construction

Use 2 mono-restriction endonuclease enzymes to digest targeted gene plasmids and vector plasmids (PGL3-basic), and synthesis the reconstructed plasmids with ligase. The products were transferred into the receptive bacteria. The monoclonal colonies were sent for sequencing identification.

-AhR overexpression plasmid construction

Designed the primers of AhR, and obtained targeted fragments by PCR amplification. Use 2 mono-restriction endonuclease enzymes to digest targeted fragments and plasmids(pcDNA3.1), and synthesis the reconstructed plasmids with ligase. The products were transferred into the receptive bacteria. The monoclonal colonies were sent for sequencing identification.

The reporter gene plasmids with Nrf2 promoter sequence were co-transfected with AhR overexpression or empty plasmid into 293 T cells. 293T cells were cultured in Dulbecco's modified Eagle's medium (DMEM) containing 10% fetal bovine serum. The cells were transiently transfected with Lipofectamine™ 3000 Reagent (Invitrogen) for 8 h. After 24 h, transfected cells were treated with ox-LDL or ox-LDL and ICA. After 24 h, luciferase activity was tested using a Dual-Luciferase Reporter System (GENECHEM) and normalized to Renilla luciferase activity.

The primer sequences of the AhR overexpression promoter were as follows: F: 5′-GCTTGGTACCGAGCTCGGATCCGCCACCATGAACAGCAGCAGC-3; R: 5′-GTTTAAACGGGCCCTCTCTCGAGTTACAGGAATCCACTGGATGTCAAATCAGG-3′; the sequences of the Nrf2 promoter were from -1457to+555.

In vivo knockdown with siRNA

Gene expression was knocked down in cells by siRNA. Genomeditech helped to design siRNA of AhR NRF2 and HO1. When cell density reached 50%–60%, HUVEC was incubated with siRNA and Lipofectamine™ 3000 Reagent for 6 h and then incubated with ECM for 24 h. After overnight incubation, cells were treated with ox-LDL and ICA. Interference efficiency was determined using qPCR.

Chromatin immunoprecipitation

The cells were cross-linked with 1% paraformaldehyde for 10 min, glycine was added and the final concentration was adjusted to 0.125M, following which cells were lysed with lysis buffer (WELLBIO) containing 1× protease inhibitor cocktail (WELLBIO)， after three washes with ice-cold. Then centrifugal cells were sheared using sonication. After centrifugation, supernatants were added to ChIP incubation buffer (WELLBIO). Ten micrograms of antibody (Invitrogen) were added to Protein A+G magnetic beads (Magna ChIPTM 16-663) and washed with ice-cold PBS containing 1% BSA, then added to the ChIP incubation buffer for immunoprecipitation at 4 ℃ overnight. The magnetic beads were washed once with low-salt wash buffer, high-salt wash buffer, and LiCl wash buffer and twice with TE buffer. The complexes were supplemented with 1% SDS, 0.1 M NaHCO3. Then 5 M NaCl was added and the immunoprecipitated chromatin was heated at 65 °C overnight to reverse the cross-linking. The DNA fragments were purified with Rnase and the final DNA fragments were analyzed using a real-time PCR kit. The following primer pairs were designed for promoter detection: NRF2 P_Pos1 left primer： GCTGAGCTTCCGAAAATCCC, and right primer: GAGAGCGCTGCCCTTATTTG; P_Pos2 left primer: GACGTGGTTTGGCTCTCG and right primer: GCGTCTGGAGCTTCACATTC; P_Pos3 left primer: ACACTCGCAACTCTTACCCT and right primer: CAAGGGACAGGTTGGAGCT P_Pos4 left primer: GCAACAGATCAACAGCTCCA and right primer: CTTCTAGTTCGGACGCGGT; P_Neg1 left primer: TAAAGCAAAAGTGGCACCCC and right primer: GCTCACACATCTCCCTCTCA; P_Neg2 left primer: CAGAGGAAGAGGCTTACTGGT and right primer: TGACAGACATGAGCCAGTGT

Statistics

Values with P < 0 .05 were considered statistically significant. Data are shown as mean values ± SEM. Statistical analysis was performed using GraphPad Prism Software Version 9 (San Diego, CA, USA). Two-tailed Student’s t-test was performed to analyze the differences between the two groups. For multiple comparisons, one-way ANOVA was used. The data in this study were obtained either from one experiment with three to six replicates or from three independent experiments.

Figure. S1.


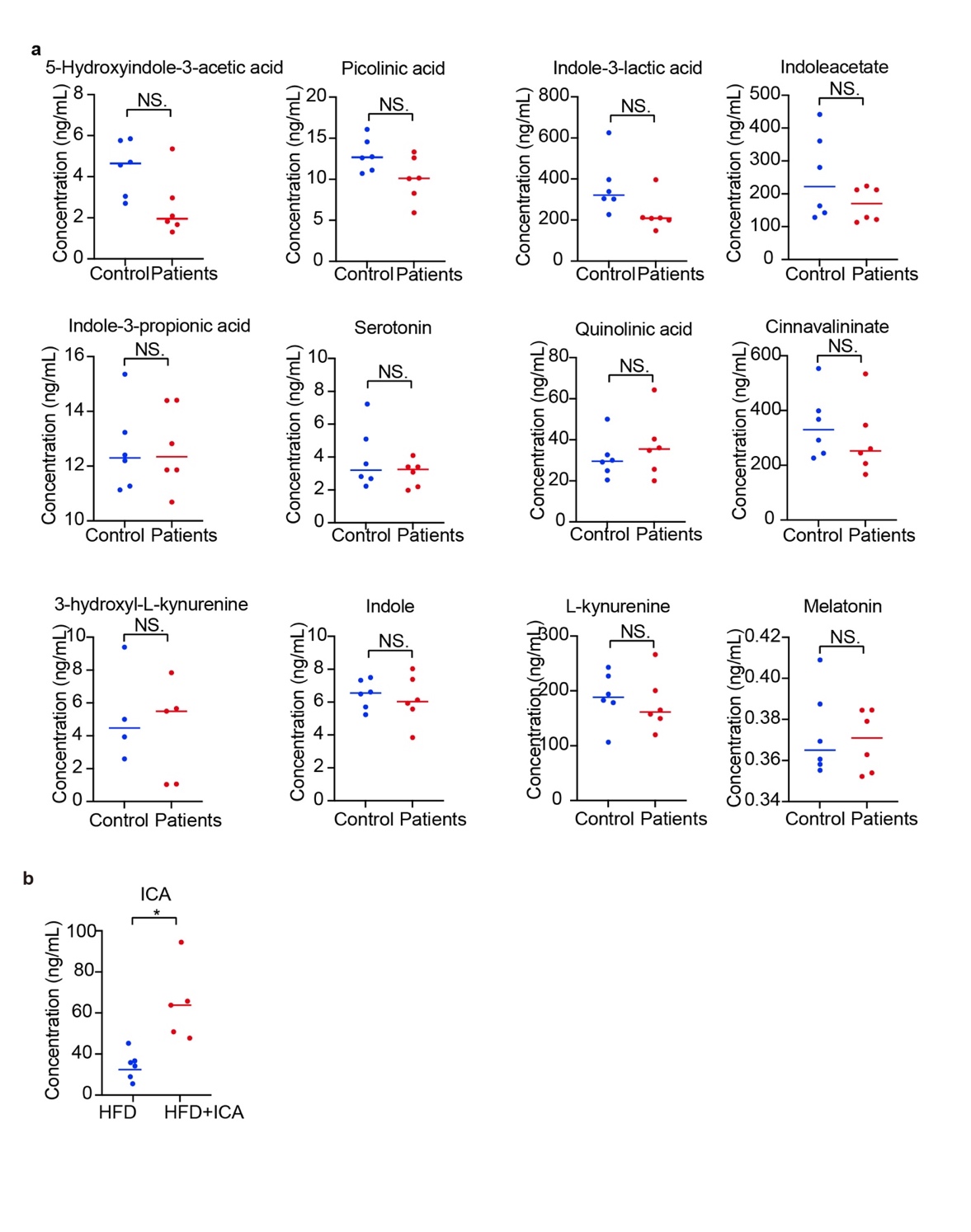


a. plasma concentration of indole derivatives in CAD patients and healthy controls. b. plasma concentration of ICA in HFD and HFD+ICA groups. Data are means±SD and were analyzed by an unpaired 2-tailed Student t test.

Figure. S2.


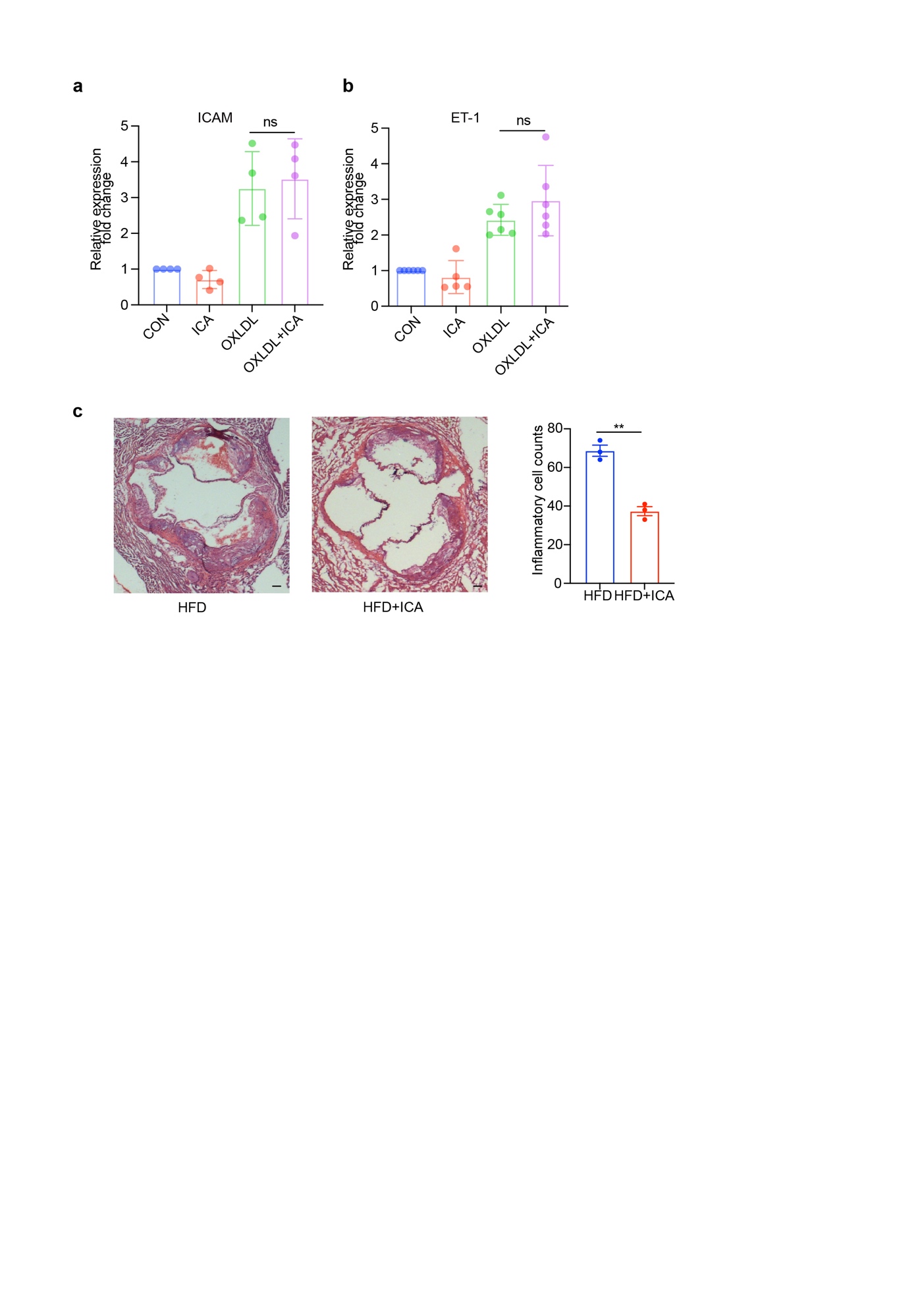


Real-time polymerase chain reaction analysis of the endothelial function associated genes (a)ICAM,and (b)ET-1 in endothelial cells treated with ox-LDL (100 mg/mL) and ICA (100 nmol) (n = 4 per group). Graphs represent the mean ± SEM from at least n = 4–6 replicates and one-way ANOVA with Tukey’s multiple comparison test. (*P<0.05, **P≤0.01, ***P≤0.001, ****P≤0.0001)(c) Representative images of HE staining of the aortic sinus lesion area (n = 10, scale bar: 100 μm). Data are presented as means ± SEM. Data were analyzed using an unpaired two-tailed Student t-test.

Figure. S3.


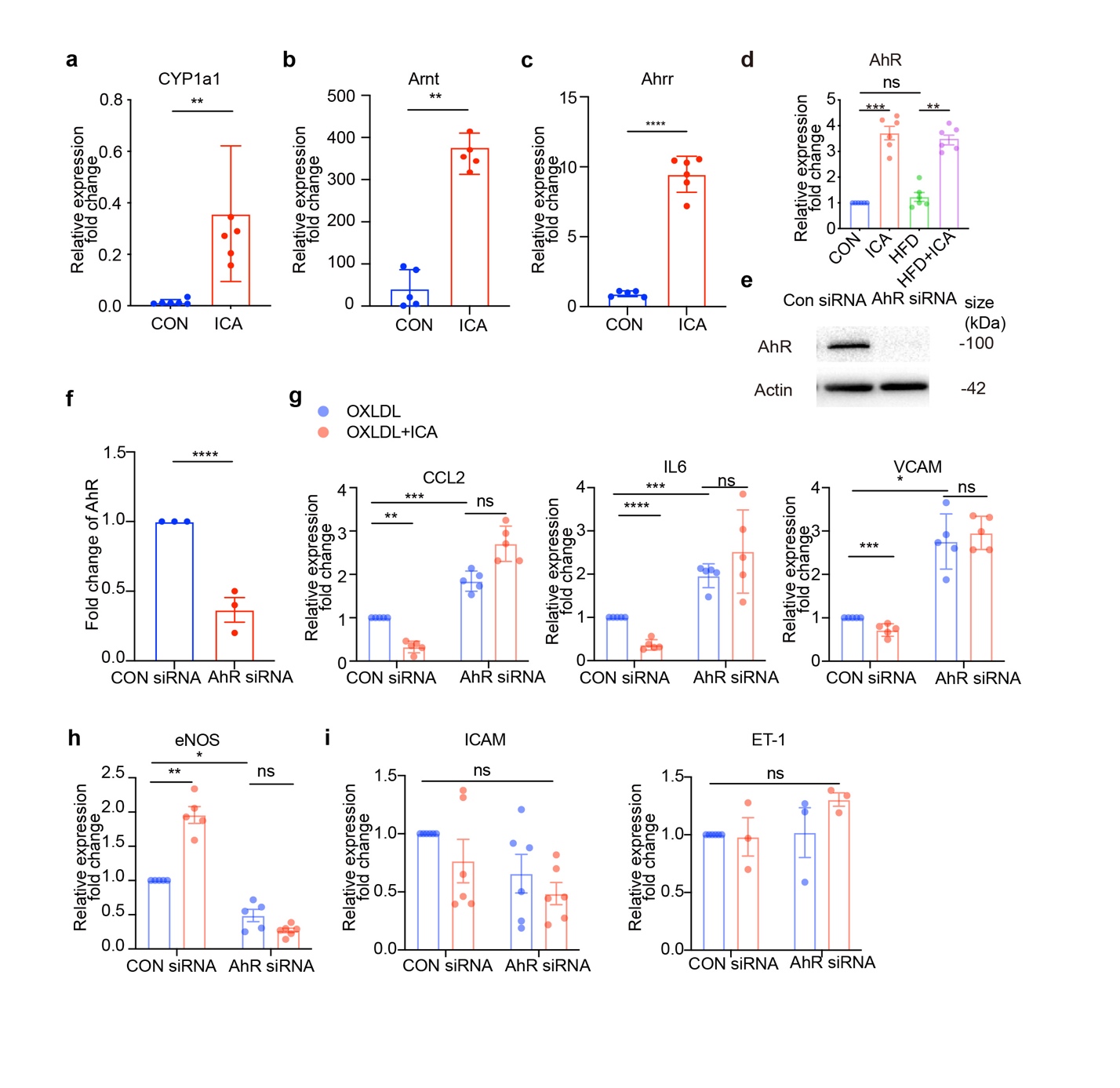


ICA prevents endothelial dysfunction and ROS through AhR. (a-c) Expression of AhR downstream genes such as CYP1a1, Arnt, and Ahrr in HUVEC treated with ICA (n = 4 per group). (d) AhR expression in mice small intestine.(e) HUVEC cells were transfected with control or AhR siRNA. AhR protein expression was determined using western blotting (n = 3 per group). (f) Quantification of AhR protein expression and the results are presented as the mean ± SEM of the fold change of control siRNA (n = 3). Statistical significance was determined using an unpaired two-tailed Student t t-test. The expression of (g) CCL2, IL6, VCAM and (h-i)eNOS, ICAM ET-1was measured using qPCR. Graphs represent the mean ± SEM from at least n = 4–6 replicates, and statistical significance was determined by two-way ANOVA with Tukey’s multiple comparison test. (*P<0.05, **P≤0.01, ***P≤0.001, ****P≤0.0001)

Figure. S4.


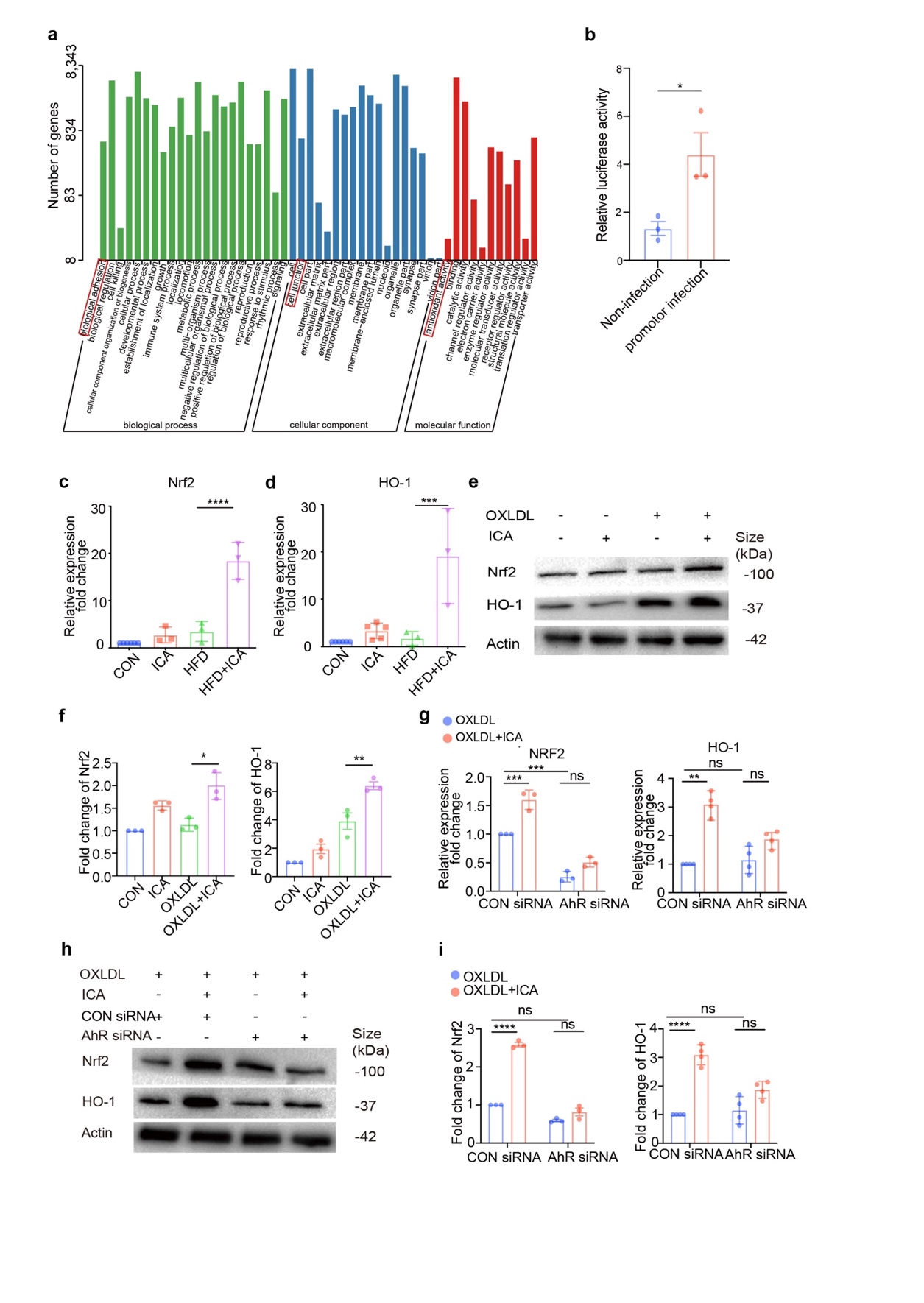


AhR regulates Nrf2 expression after activation by ICA. (a) GO enrichment and binding site analysis. AhR binds to the promoter region of Nrf2, as supported by the dual-luciferase reporter system (b). 293T cells were transferred with PGL3-Nrf2-promoter plasmids and PGL3-AhR plasmids. Non-infection group is the control. Data are presented as mean ± SEM, and statistical significance was determined using an unpaired two-tailed Student t-test. (c) RNA levels of Nrf2 and its downstream genes, (d) HO-1 in endothelial cells treated with ox-LDL and ICA. Data are presented as mean ± SEM, and P values were determined by one-way ANOVA and Tukey’s multiple comparison test. (e) Protein expression of Nrf2 and HO-1 was determined using western blotting. (f) Quantification of Nrf2 and HO-1 protein levels are presented as mean ± SEM (n = 3). P values were determined by one-way ANOVA and Tukey’s multiple comparison test. The expression of Nrf2 and HO-1 after silencing AhR was measured by qPCR (g) and western blotting (h-i), respectively. Graphs represent the means ± SEM (n = 3) and two-way ANOVA with Tukey’s multiple comparison test. (*P<0.05, **P≤0.01, ***P≤0.001, ****P≤0.0001)

Figure. S5.


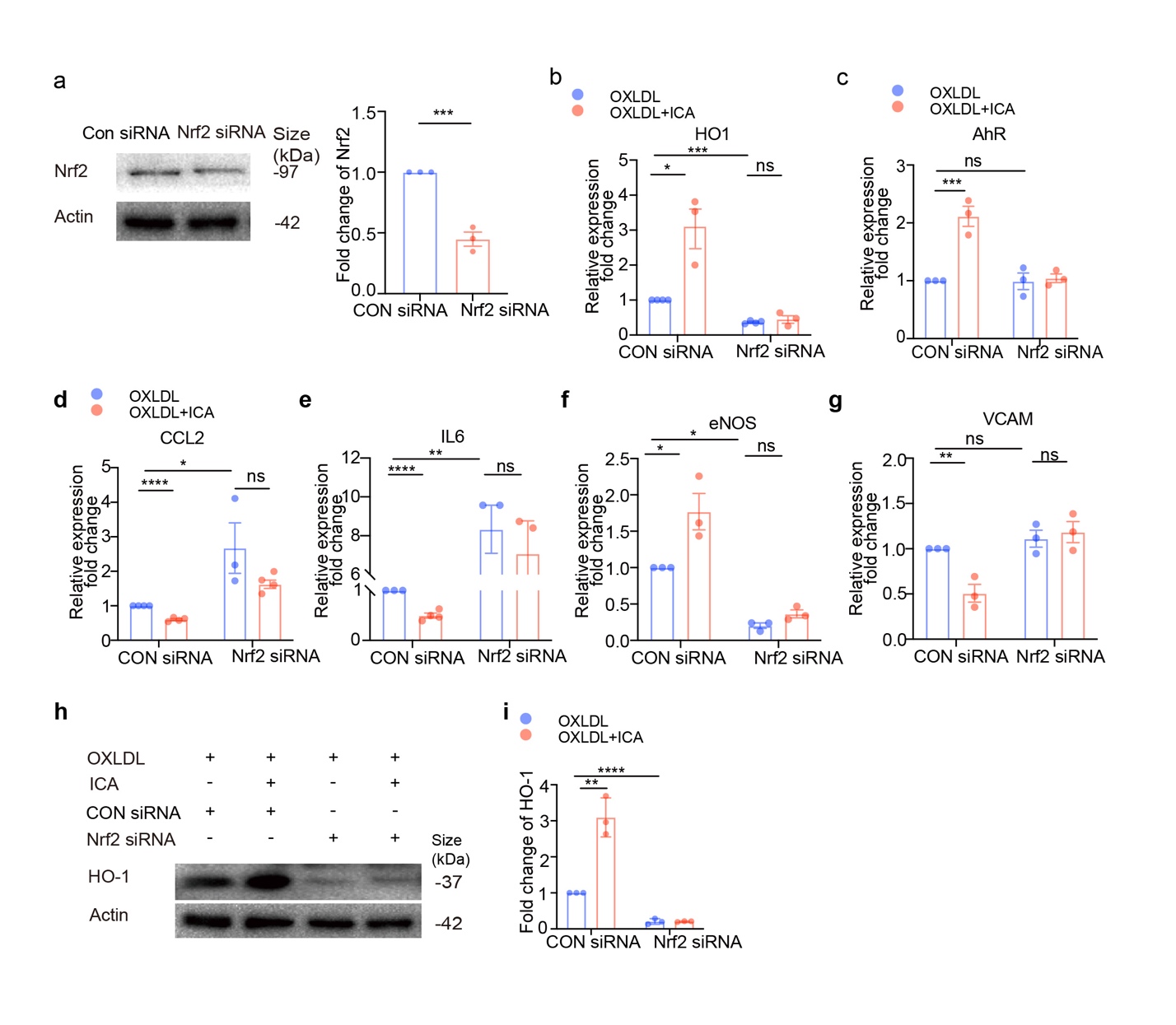


AhR regulates Nrf2 to improve endothelial function and decreases cellular ROS level. (a) HUVEC cells were transfected with control or Nrf2 siRNA. AhR protein expression was determined using western blotting (n = 3 per group). (b) Quantification of Nrf2 protein expression and the results are presented as the mean ± SEM of the fold change of control siRNA. Statistical significance was determined using unpaired two-tailed Student t-test. From (c) to (g), total RNA isolated from treated cells was assessed for the mRNA levels of HO-1, AhR, CCL2, IL6, eNOS, and VCAM using quantitative real-time polymerase chain reaction. Graphs represent the mean ± SEM from at least n = 4–6 replicates, and statistical significance was determined by two-way ANOVA and Tukey’s multiple comparison test. (*P<0.05, **P≤0.01, ***P≤0.001, ****P≤0.0001)

Figure. S6.


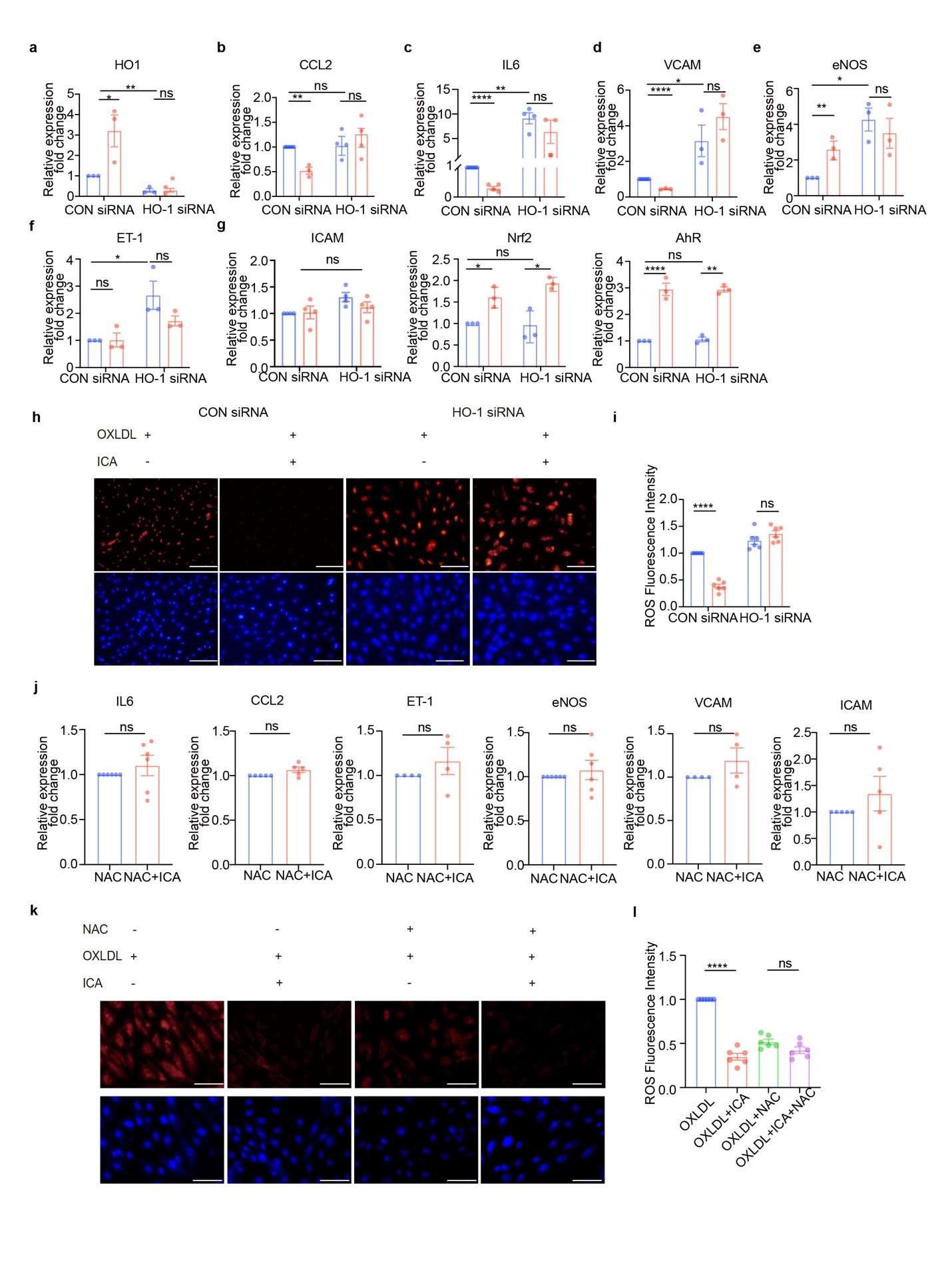


ICA protects against endothelial dysfunction and ROS through the AhR-Nrf2-HO-1 axis. From (a) to (g), total RNA isolated from treated cells was assessed for the mRNA levels of HO-1, ET-1, IL6, VCAM, eNOS, CCL2, ICAM, Nrf2 and AhR using quantitative real-time polymerase chain reaction. Graphs represent the mean ± SEM from at least n = 4–6 replicates, and statistical significance was determined by two-way ANOVA and Tukey’s multiple comparison test. (h) DHE and DAPI staining of HUVEC cells transfected with control siRNA or HO-1 siRNA treated with ox-LDL and ICA, respectively (n = 6, scale bar: 100 μm). (i) Quantification of the DHE fluorescence intensity. Data are presented as mean ± SEM, and P values were determined by two-way ANOVA and Tukey’s multiple comparison test. (j) Both groups were treated with ox-LDL and NAC, and total RNA was isolated from ICA-treated and non-treated cells. The mRNA levels of IL6, CCL2, ET-1, eNOS, VCAM, and ICAM were assessed using quantitative real-time polymerase chain reaction. Graphs represent the mean ± SEM from at least n = 4–6 replicates, and statistical significance was determined using an unpaired two-tailed Student t-test. (k) Quantification of the DHE fluorescence intensity (n = 6, scale bar: 75 μm). Data are presented as mean ± SEM, and P values were determined by two-way ANOVA and Tukey’s multiple comparison test. (*P < 0.05, **P ≤ 0.01, ***P≤0.001, ****P≤0.0001)

Figure. S7.

**
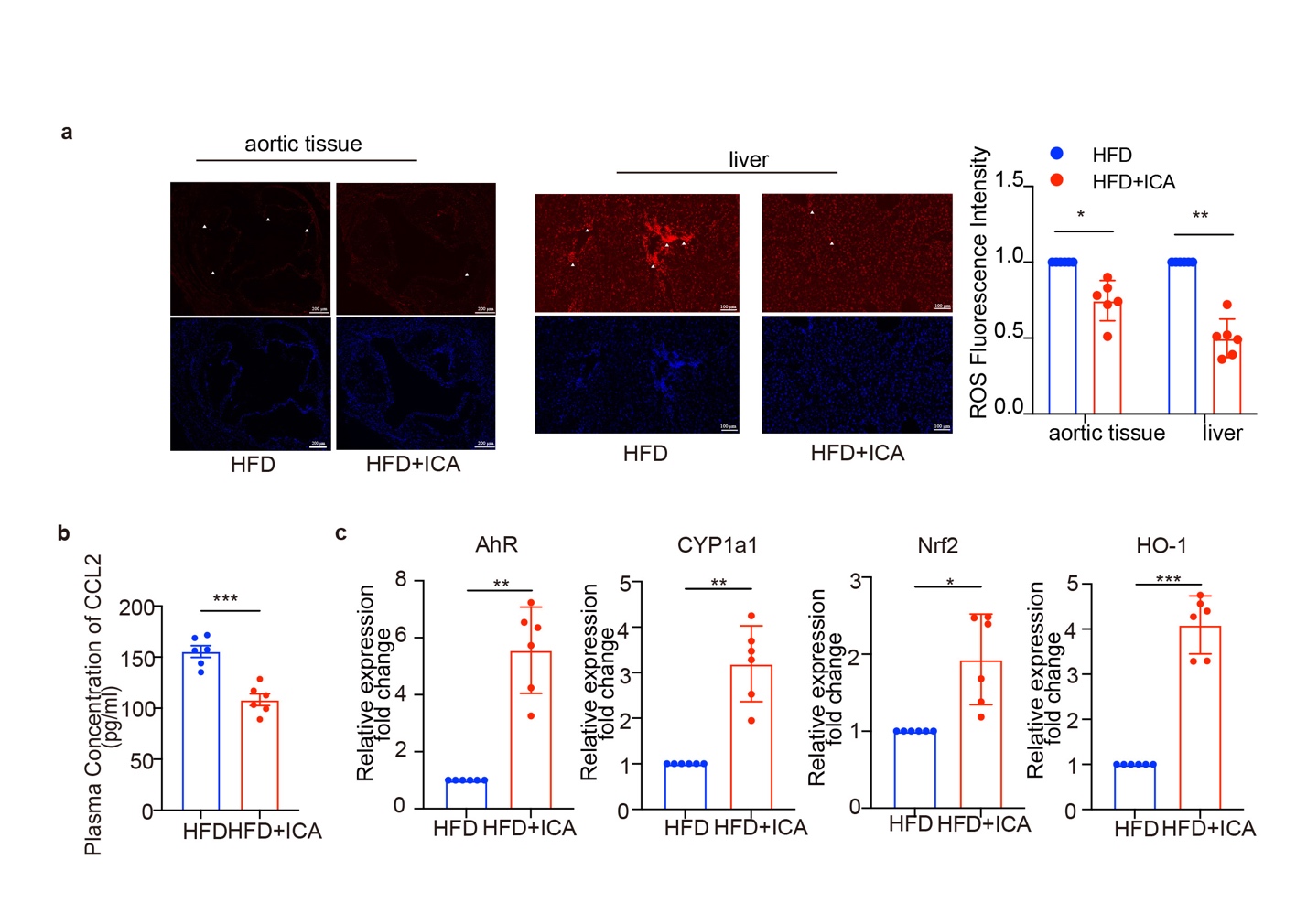
**

(a) DHE and DAPI staining of aortic root and live. (n = 6, scale bar: 200 μm, scale bar: 100 μm). Quantification of the DHE fluorescence intensity. Data are presented as mean ± SEM, and P values were determined by an unpaired two-tailed Student t-test. (b) The plasma concentration of CCL2 (n = 6) in ApoE-/- mice fed with HFD or with HFD+ICA. (c) Expression of AhR, CYP1a1, Nrf2 and HO-1 in aortic endothelial cells from ApoE-/- mice fed with HFD or with HFD+ICA. (n = 6) Graphs represent the mean ± SEM and were P values determined by an unpaired two-tailed Student t-test. (*P < 0.05, **P ≤ 0.01, ***P≤0.001, ****P≤0.0001)

Table S1.


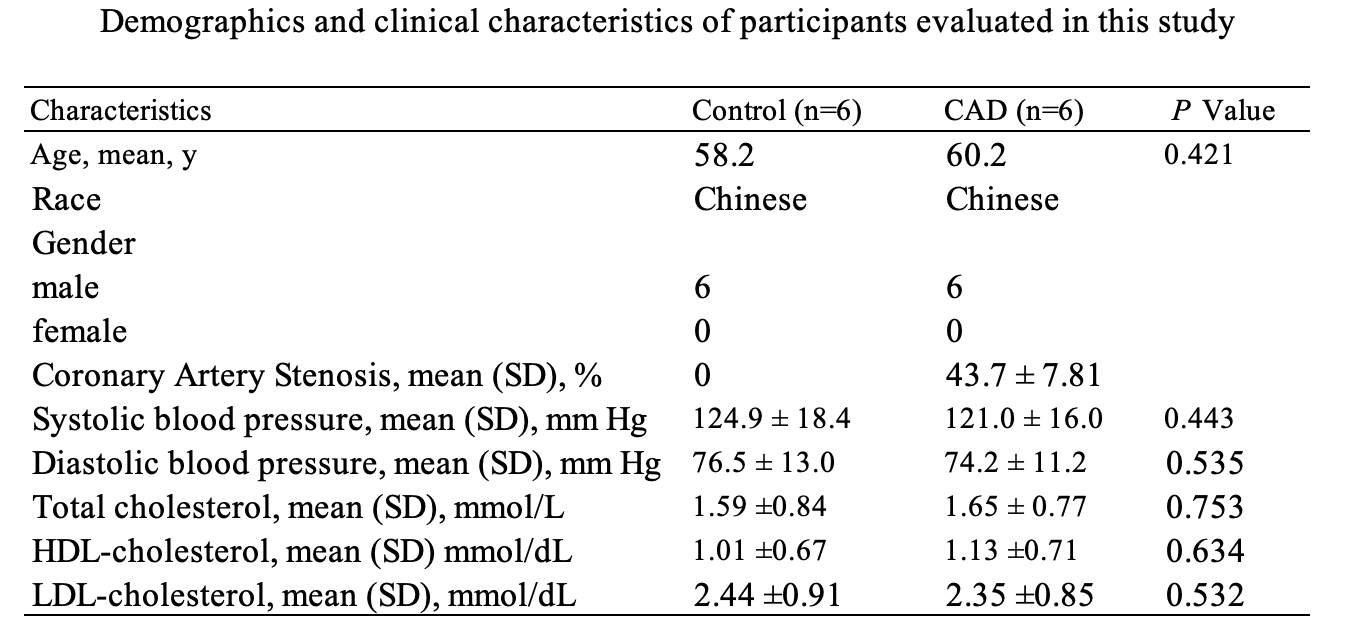


The demographics and clinical characteristics of participants. Both groups have 6 samples. Patients with over 40% coronary artery stenosis are included as CAD patients. CAD patients with other major disease are excluded for example: cancer.
