# Supplementary material for: Gut microbe-derived metabolite indole-3-carboxaldehyde alleviates atherosclerosis
Source: Signal Transduct Target Ther. 2023 Oct 4;8:378. doi: 10.1038/s41392-023-01613-2 (PMC10547776; doi:10.1038/s41392-023-01613-2)

S3.e：FROM lane1 to lane 8：

CON SIRNA1；CON SIRNA 2；CON SIRNA3；CON SIRNA4；CON SIRNA5；CON SIRNA6

AhR SIRNA1；AhR SIRNA2


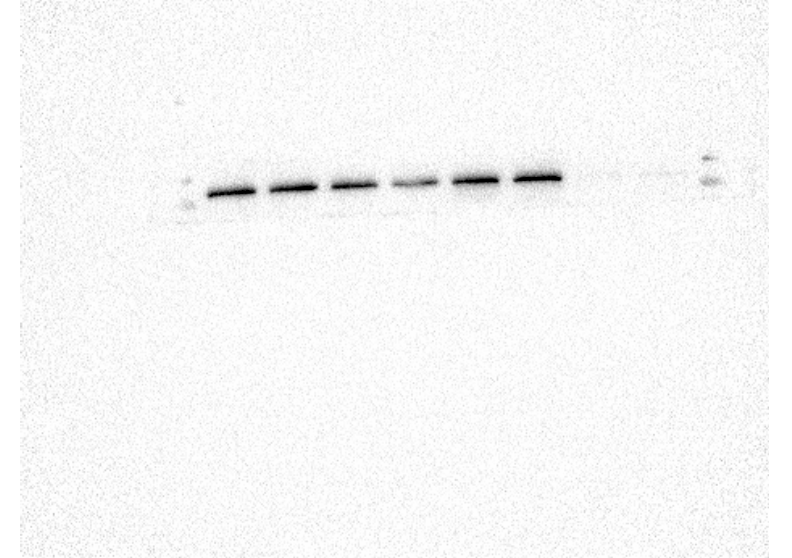


AhR -100 KDa


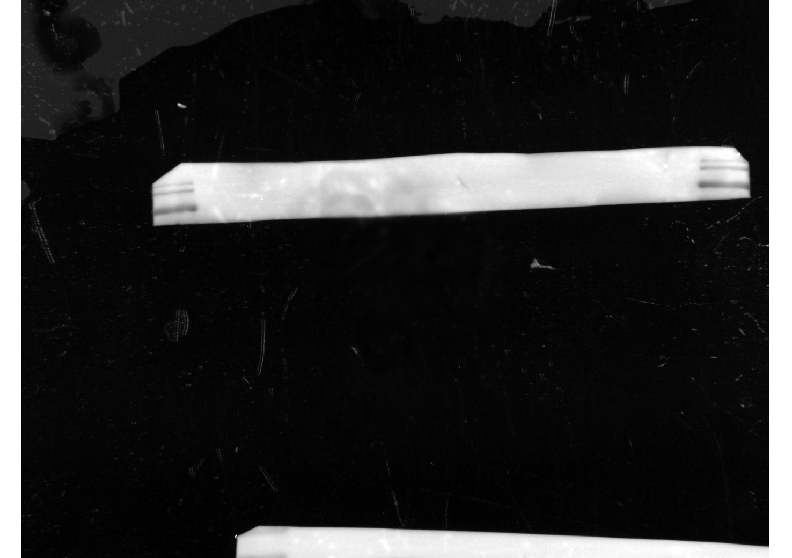


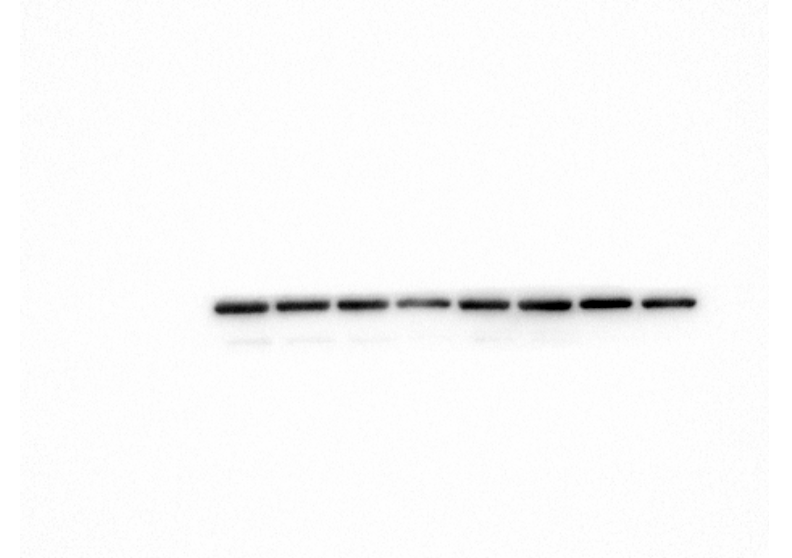


actin-42 KDa


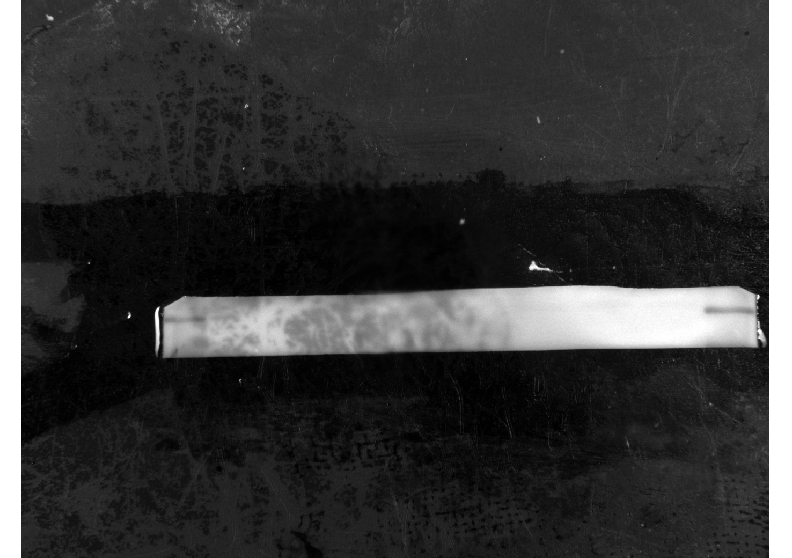

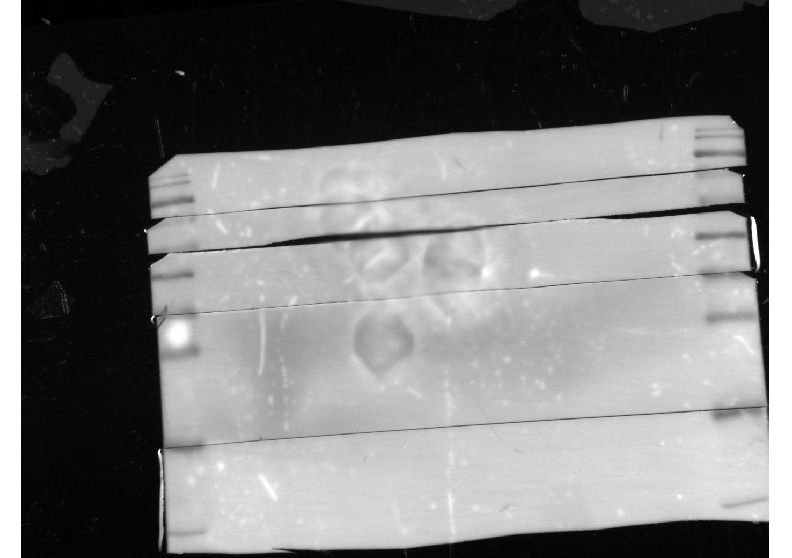


S4.e：FROM lane1 to lane 4：

Con；ICA;OXLDL;OXLDL+ICA


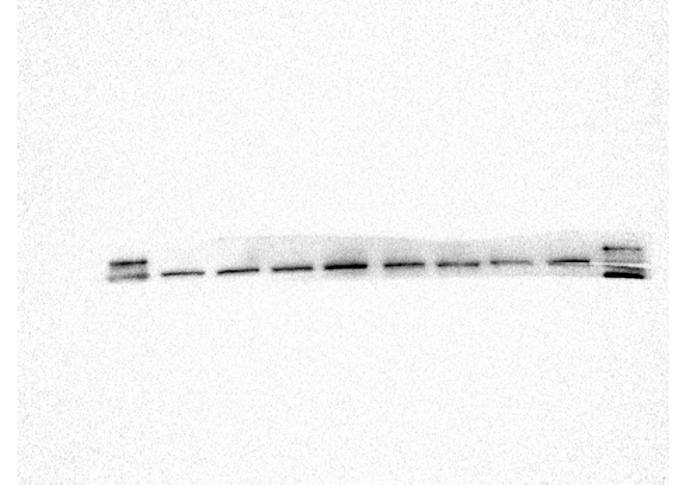


NRF2 -100 KDa


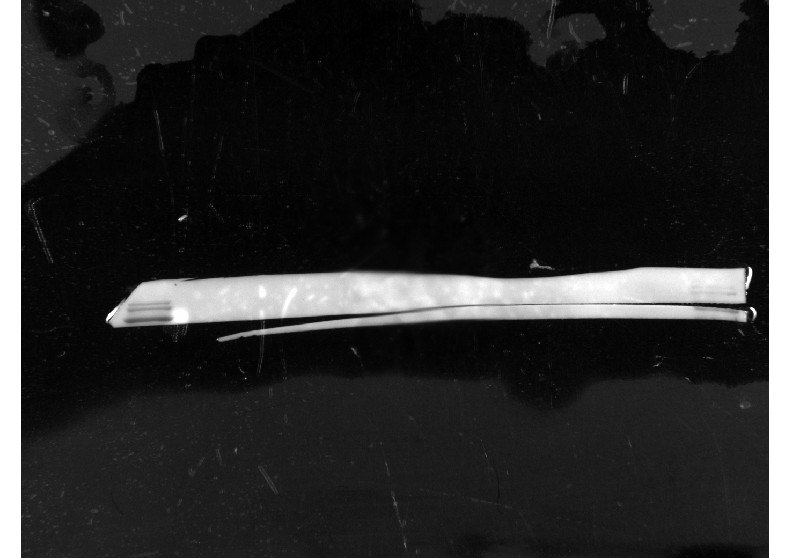


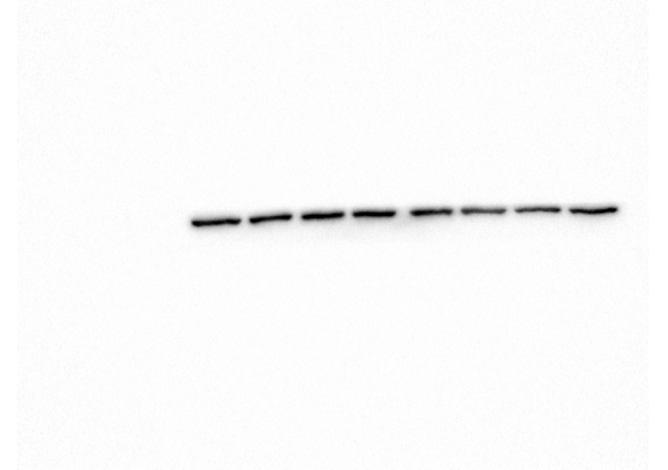


actin-42 KDa


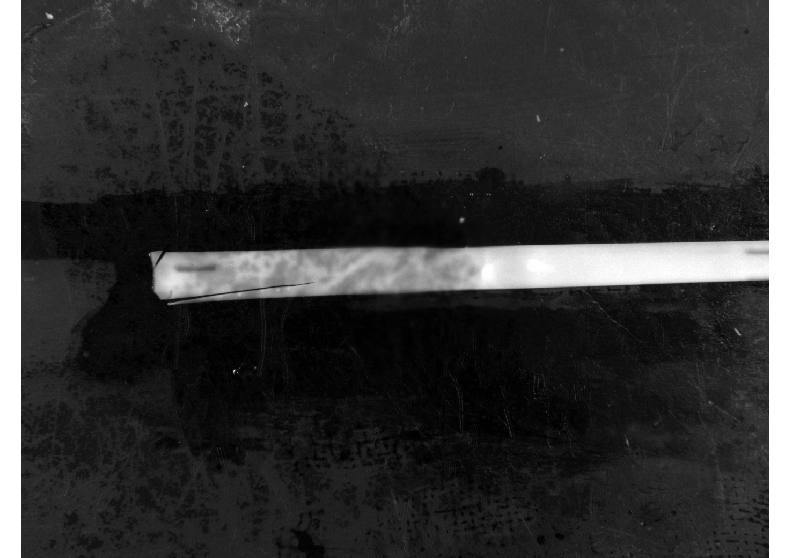

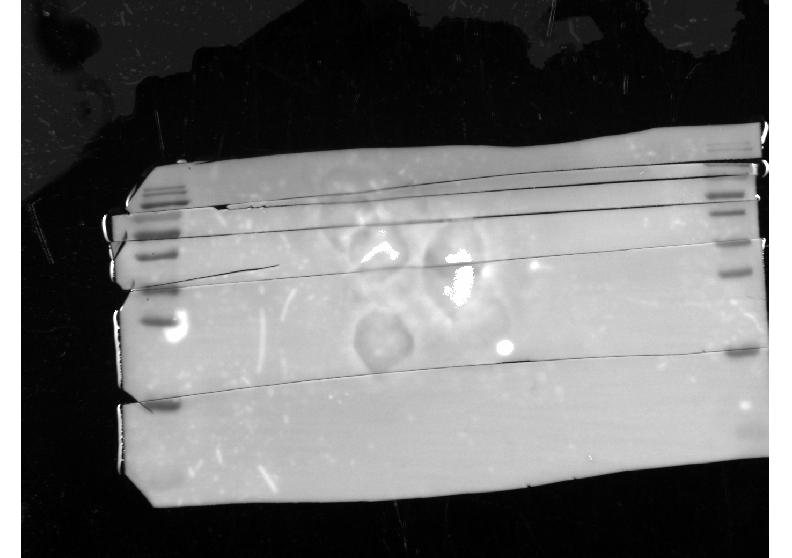


S4.e：FROM lane5 to lane 8：

ConSirna+oxldli；ConSirna+oxldl+ICA;

Ahr sirna+OXLDL; Ahr sirna+OXLDL+ICA


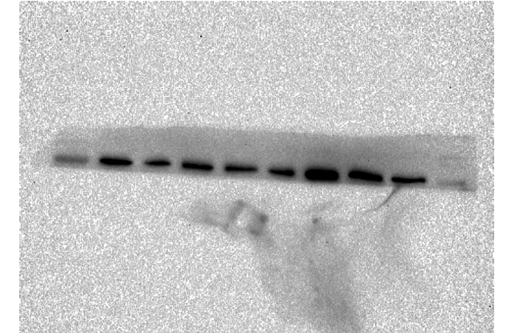


NRF2 -100 KDa


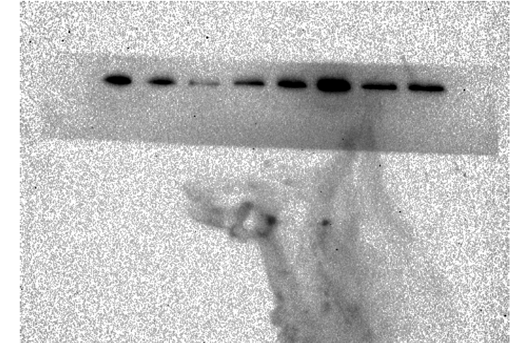


Ho-1 -37 KDa


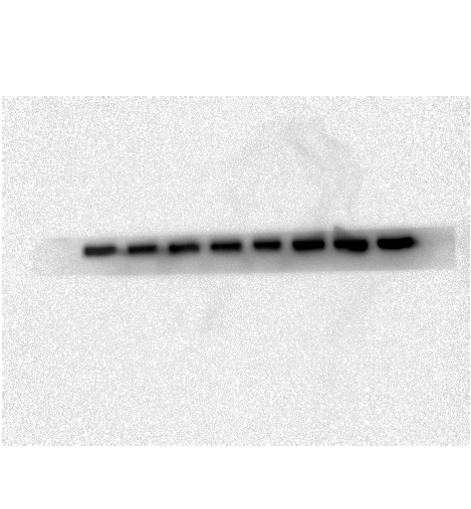


actin -42 KDa

S5h
FROM lane1 to lane 4：

ConSirna+oxldli；ConSirna+oxldl+ICA;

Nrf2 sirna+OXLDL;nrf2 sirna+OXLDL+ICA


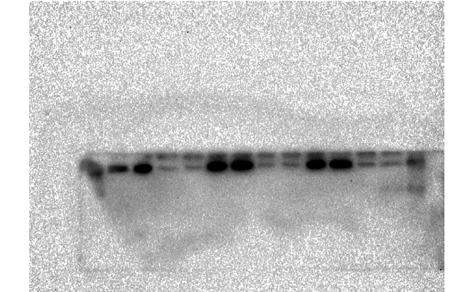

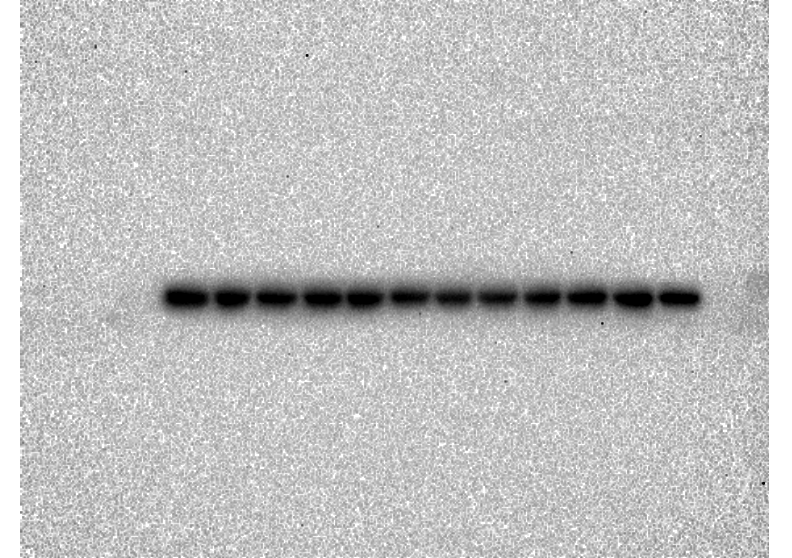


actin -42 KDa

Ho-1 -37 KDa


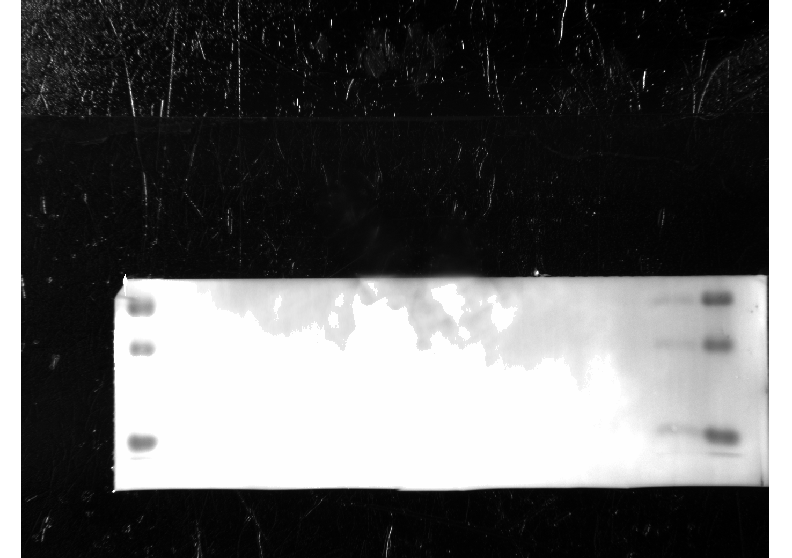

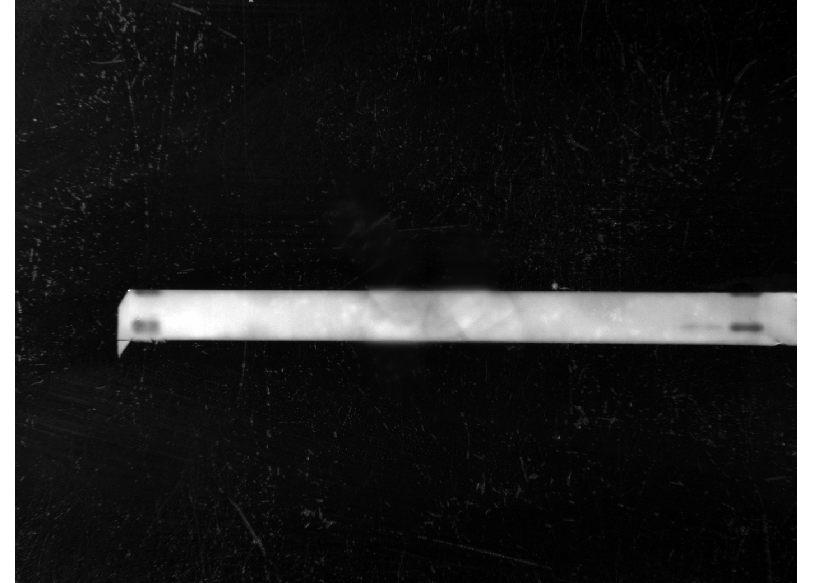


S5a

FROM lane5 to lane 6：

ConSirna；ConSirna

Nrf2 sirna; Nrf2 sirna


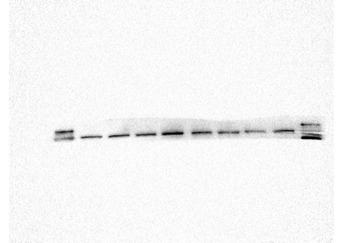

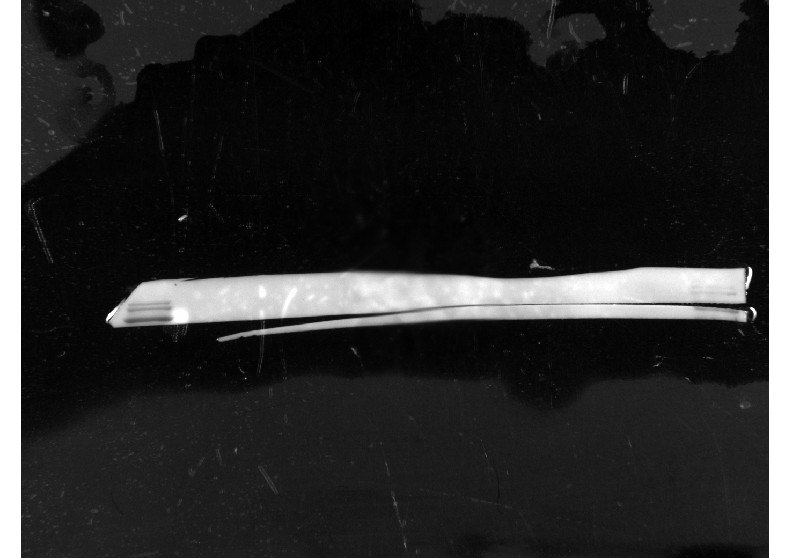


NRF2 -100 KDa


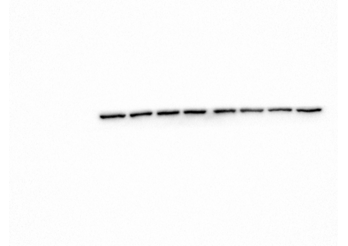

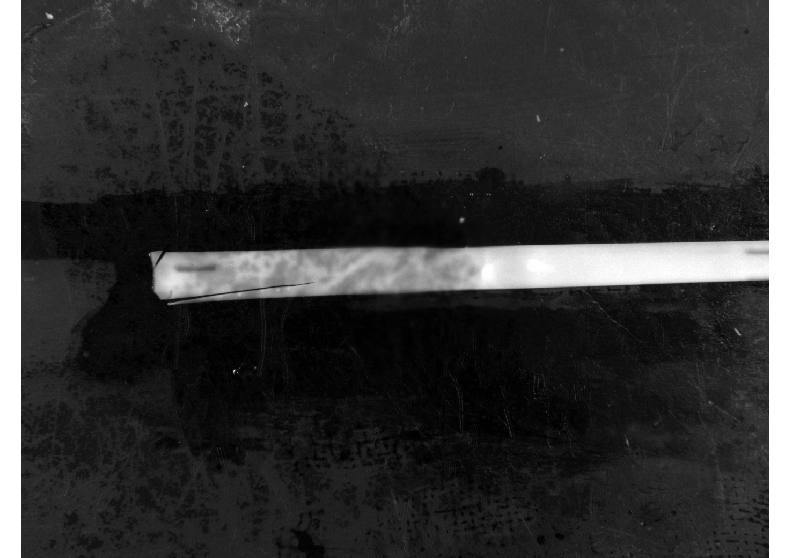


actin -42 KDa


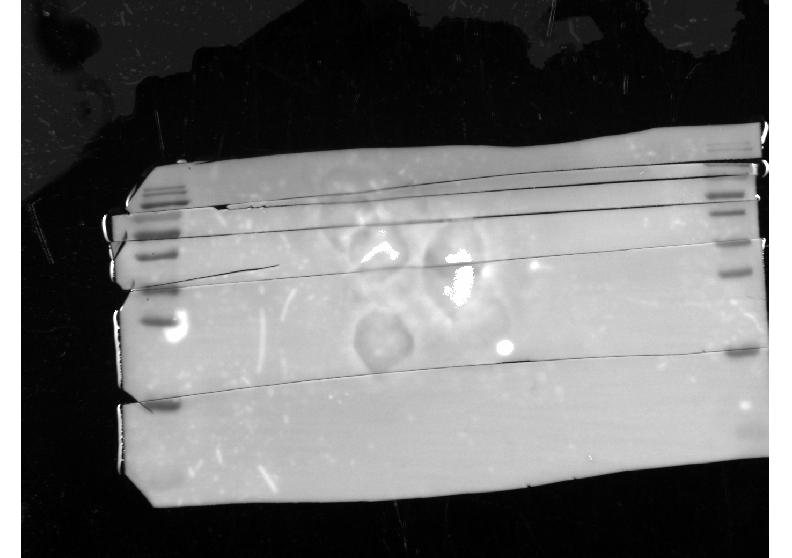

Supplement: Supplementary file 2 — Gut microbe-derived metabolite indole-3-carboxaldehyde alleviates atherosclerosis [file 41392_2023_1613_MOESM2_ESM.docx]
